# Investigating the role of the foveal cortex in peripheral object discrimination

**DOI:** 10.1038/s41598-022-23720-w

**Published:** 2022-11-19

**Authors:** Giulio Contemori, Carolina Maria Oletto, Roberta Cessa, Elena Marini, Luca Ronconi, Luca Battaglini, Marco Bertamini

**Affiliations:** 1 Department of General Psychology, University of Padova, 35131 Padova, Italy; 2 School of Psychology, Vita-Salute San Raffaele University, 20132 Milan, Italy; 3 Division of Neuroscience, IRCCS San Raffaele Scientific Institute, 20132 Milan, Italy; 4 Department of Psychology, University of Liverpool, Liverpool, UK

**Keywords:** Cognitive neuroscience, Psychology

## Abstract

Peripheral object discrimination is hindered by a central dynamic mask presented between 150 and 300 ms after stimulus onset. The mask is thought to interfere with task-relevant feedback coming from higher visual areas to the foveal cortex in V1. Fan et al. (2016) supported this hypothesis by showing that the effect of mask can be further delayed if the task requires mental manipulation of the peripheral target. The main purpose of this study was to better characterize the temporal dynamics of foveal feedback. Specifically, in two experiments we have shown that (1) the effect of foveal noise mask is sufficiently robust to be replicated in an online data collection (2) in addition to a change in sensitivity the mask affects also the criterion, which becomes more conservative; (3) the expected dipper function for sensitivity approximates a quartic with a global minimum at 94 ms, while the best fit for criterion is a quintic with a global maximum at 174 ms; (4) the power spectrum analysis of perceptual oscillations in sensitivity data shows a cyclic effect of mask at 3 and 12 Hz. Overall, our results show that foveal noise affects sensitivity in a cyclic manner, with a global dip emerging earlier than previously found. The noise also affects the response bias, even though with a different temporal profile. We, therefore, suggest that foveal noise acts on two distinct feedback mechanisms, a faster perceptual feedback followed by a slower cognitive feedback.

## Introduction

There are situations in which we must recognize objects in the periphery of the visual field without — or before — being able to move our eyes. In some cases, we can successfully identify an object despite the obvious limits of peripheral vision. To understand how this happens, we need to move beyond a classical retinotopic framework of vision and consider scene perception as the result of processes that integrate input coming at different times and from different parts of the visual field^2,3^.

According to the traditional view, visual object recognition relies primarily on a hierarchical feedforward model in which the early processing stages are strongly retinotopic^4,5^. More recently, evidence has shown that visual processing is more flexible. In 2008 the seminal work of Williams et al.^6^, showed an involvement of the foveal retinotopic cortex in the processing of information presented more than five degrees outside the fovea. They found a task-related activation in the foveal cortex for stimuli presented at different peripheral locations. The authors argued that the foveal retinotopic cortex was recruited by feedback signals from higher-level visual areas to participate in object recognition as an auxiliary computational module^6^.

Five years later, Chambers et al.^7^ provided causal evidence of the role of this foveal feedback for peripherally presented object discrimination. They showed that disrupting foveal processing with a transcranial magnetic stimulation (TMS) pulse at the posterior calcarine site around 350 ms after target presentation affected peripheral, but not foveal, object discrimination. This result was further corroborated by two studies that, despite different paradigms, found compatible results, showing that performance in visual tasks declines when the early visual cortex is stimulated with a TMS pulse between 90 and 320 ms^8,9^. It is critical to note that this timing is not consistent with a disruption of feedforward processing but is consistent with a late feedback signal^7^. Further support for the peripheral-to-foveal feedback hypothesis comes from behavioral studies in which interference was produced by a subsequent mask (for a review, see Stewart et al.^3^). Mask disruption on peripheral discrimination occurred in a specific time window, which ranged from 117^10–12^ to 300 ms^1,13^. The importance of feedback in early visual processing was known prior to these studies, but it was thought to represent a predictive mechanism that would support feedforward visual processing within a rigid retinotopic organisation^14^. The discovery that this type of feedback is position invariant suggests otherwise^6^.

One possibility is that this feedback is necessary for processing fine details in the peripheral visual field^10,12^. Another hypothesis is that this feedback is preparatory to foveation and is therefore a by-product of saccade planning^15^. In support of this second hypothesis, Fan et al.^1^ showed that the foveal noise mask was not effective when participants made a saccade away from the peripheral objects. However, they also showed that when a mental operation (mental rotation) was required before performing the discrimination task, the disruptive effect of foveal mask presentation was delayed. If the sole purpose of foveal feedback was to provide a prime to the foveation, its timing would be bound to saccadic preparation and would not change according to the mental manipulation of the stimulus. Furthermore, if this feedback was predictive, one could expect an effect on every peripheral task independently of task difficulty. The disruptive effect of the mask instead is specific to challenging tasks that require fine object discrimination^6,13^. Finally, since we can foveate only one object at a time, in a peripheral comparison task we should expect little or no effect of mask since only information from one of the two simultaneous targets could be brought to the fovea. The fact that the noise mask also affects performance in comparison tasks shows that information carried in the fovea is important for decision making and suggests that it may be necessary to mentally manipulate peripheral objects rather than to program a saccade^1^. In this regard, the foveal feedback could be part of an imaginative system that exploits low-level visual areas as a substrate for persistence^16^ and manipulation^17,18^ of task-related visual information.

It is well known that there are variations in the oscillatory brain activity that affect performance during visual tasks, with different frequencies that are linked to specific, perceptual, attentional, and cognitive processes^19–21^. Recent studies have shown that alpha oscillations in the visual cortex would be a correlate of late feedback activity required for conscious perception^22–24^. Wilming et al.^23^ found evidence that, during perceptual decision-making, endogenous information regarding perceptual choice was related to the power of the alpha band. Furthermore, a recent study in mice showed that the same neural population in V1 is involved in both feedforward and feedback, but at different time intervals exclusively^25^.

It seems possible that there is a pattern of temporal alternation between feedback and feedforward and that the same neural population is involved in both but with different timing. The cyclic alternation of feedback and feedforward would require retinotopic neurons in the fovea to switch between internally and externally oriented processing according to a specific timing that we expect to be the individual alpha rhythm^23,25^. We hypothesize that such neurons are cyclically recruited via endogenous feedback to contribute to the mental representation of the visual stimulus, and thus to perceptual decision-making. This feedback could be part of a larger endogenous imaging system, involving the occipital, parietal, and frontal brain areas, underlying the ability of visual imagination^26^. If it were true that the mask interferes with a cyclic feedback process, it would be reasonable to expect oscillatory variations in the mask effect as well. Unfortunately, the range of stimulus onset asynchronies (SOAs) used in the previous studies (see Stewart et al.^3^ for a review) is not dense enough to allow for a proper characterization of the precise timing of the foveal mask effect and potential rhythmic fluctuations in perception that would allow testing this hypothesis.

To better characterize the temporal dynamics and origin of the foveal feedback, we planned two experiments based on the paradigm tested by Fan and colleagues in 2016^1^, which is in turn derived from the paradigm used in Williams’^6^ pioneering study.

In experiment 1, we tried to replicate the results of the main experiment in Fan et al.^1^. Until now, studies that have investigated foveal feedback have used small samples (N = 11 in Fan et al.^1^) of super-trained subjects. Here, we tested naïf participants through an online task. By doing so, we aimed to assess the effect size in the general population. In addition, contrary to previous literature, we analyzed not only how the sensitivity varies as a function of the SOA, but also the variation in the criterion. None of the previous behavioral studies analyzed the effects of mask on criterion, while in their TMS study Chambers and colleagues show a shift in criterion becoming more conservative in an expanded time window that does not perfectly mirror the effect on d’^7^. Indeed, given that the foveal feedback could also be used during the decision-making stage, we should find an alteration in the criterion in a specific direction. If the fovea is recruited as an additional computational module to resolve stimulus details, then the presence of a foveal mask may compromise details processing and induce subjects to take a more conservative approach.

In experiment 2, the aim was to have a finer analysis of the timing of the dip function. To do this, we tested the same experimental paradigm in the laboratory, but this time increasing the number of SOAs (from 5 to 60), testing the effect of the foveal mask from zero to half a second every 8.33 milliseconds. By implementing this ’dense sampling’ methodology^19,27–33^, we wanted to implement a virtually continuous sampling of the mask effect, which would not only lead to a much better characterization of the timing of the main dip, but would also be a way to test for the presence of foveal feedback related perceptual oscillations, i.e. rhythmic variations in behavioral responses reflecting an underlying neural mechanism that is cyclic (oscillatory) in nature^29,32^.

## Experiment 1

In this experiment, we tried to replicate the main finding from Fan et al.^1^. In the original work, the authors tested a small group of highly trained participants in a well-controlled laboratory environment with a large number of trials. In contrast, we tested a larger number of naïve participants and a smaller number of trials. In addition to a conceptual replication of what was done previously, we set out to study not only changes in sensitivity, but also changes in criterion due to the presence of a foveal mask. If the function of foveal feedback were simply to convey anticipatory priming to the foveal cortex, there would be no reason to expect a deviation of the response criterion. On the contrary, if the foveal cortex is recruited with the role of a high-functioning visual sketchpad, then the introduction of random noise with the right timing could shift the criterion in a more conservative direction. Failure to mentally represent one or both peripheral stimuli could lead to judgment bias, causing them to be perceived as different even when they are not.

### Participants

A total of 56 volunteers (34 females) participated in the experiment. The age range was 16–63 years. All subjects had a normal or corrected-to-normal vision. The participants were unaware of the purpose of the study and were contacted by email among the non academic acquaintances of the investigators. To ensure that the task was performed reliably, only those with an overall average sensitivity (d’) of at least 0.7 were included in the study. All participants read and accepted the informed consent before the experiments. The procedures were performed in accordance with the guidelines of the Ethics Committee for Psychological Research of the University of Padua, from which they were approved (prot. 4793), and in accordance with the Declaration of Helsinki.

### Apparatus

The study was built using PsychoPy3^34^ and carried out remotely using the Pavlovia server. This required the participants to perform the experiment on their own personal computers. Participants were instructed to stand at a viewing distance of 57 cm from the screen. A monitor no smaller than 11” and with a refresh rate of 60 Hz was required. At the beginning of the experiment, participants were asked to resize a rectangle on the screen to match the size of a credit card. In this way we could set a scaling factor for the stimuli, independent of the monitor resolution. The temporal frequency of the monitor was recorded during the experiment and all participants with monitor refresh rate different than 60 Hz were discarded from further analysis.

### Experimental design

The experimental design was based on the original experiment by Fan et al. In total, there were 24 different conditions according to a factorial design 2 × 2 × 6. The factors involved were the type of target (same/different), the positioning of the stimuli on the screen (45° or 135° diagonal), and the temporal distance between the targets and the foveal mask (no-noise, 50, 150, 250, 350, 450 ms).

In the original experiment, 11 subjects participated, performing 2.688 trials each (excluding practice). Such a high number of trials per subject would cause a high drop rate in remote data collection. To approximate the overall statistical power of the Fan study while reducing the total duration, we increased the number of subjects to 56 who met the inclusion criteria. Eight participants who had an overall sensitivity index (d’) lower than 0.7 were excluded from the sample and replaced. Each subject repeated each condition 22 times, for an individual total of 528 trials subdivided into two blocks.

Before starting the test, to familiarize participants with the stimuli and the task, each subject watched a video in which the task and the stimuli were described in detail. The task consisted of comparing the two peripheral stimuli and making a same/different judgment by pressing one of the two response keys on the keyboard. Before the actual experimental block, subjects had to complete a practice block in which feedback was given after each response. The practice block consisted of 24 $\times$ 11 trials for a total of 264 trials and lasted about 10 minutes. Although the amount of practice may seem high, our participants were less experienced than those in the original study. In fact, the authors reported that “subjects performed several training sessions prior to the experiment” (Fan et al.^1^, Supporting Information, p. 1).

### Stimuli and procedure

**Figure 1:**
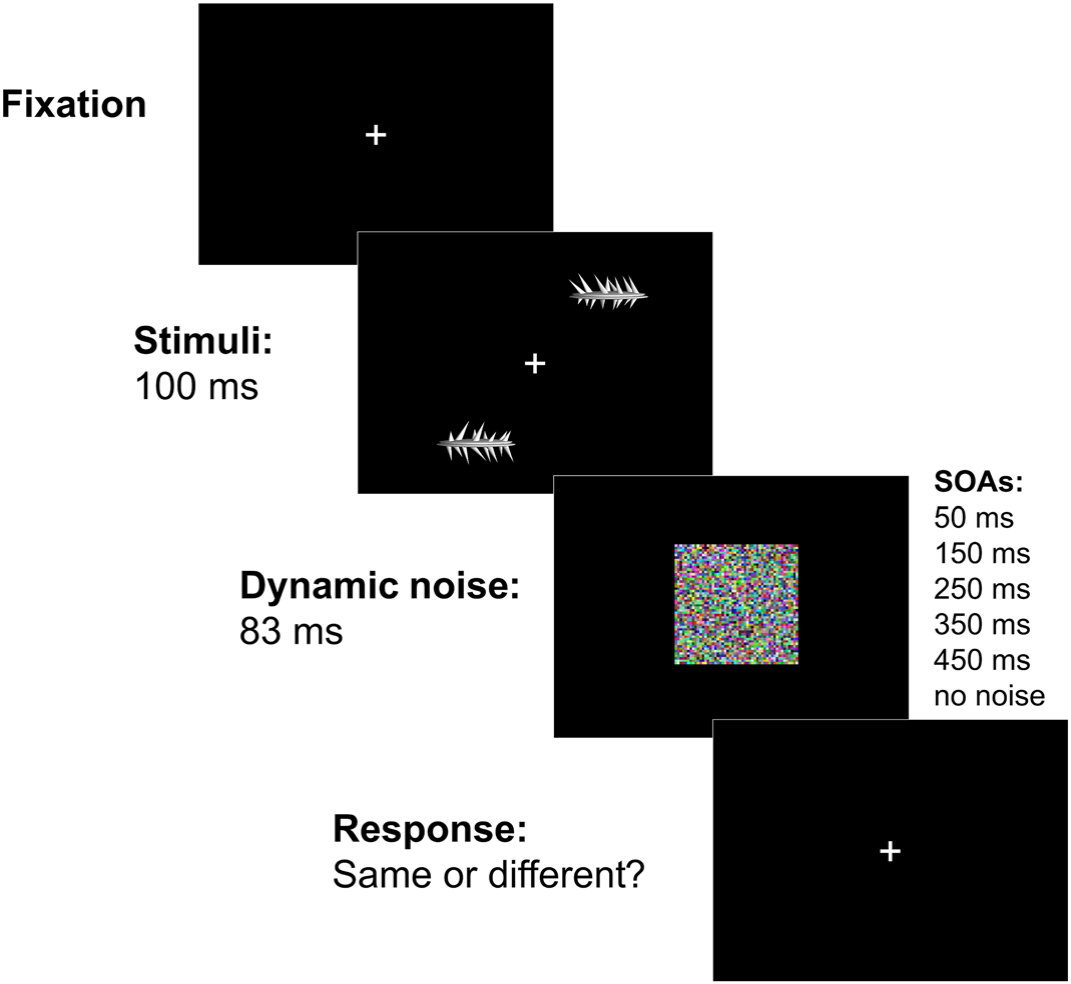
Schematic of a trial in the experiment. Two spikey objects were presented for 100 ms in the periphery of the visual field in diagonally opposite quadrants. A dynamic noise mask patch appeared in fovea for 83 ms at 5 different SOAs: 50, 150, 250, 350 and 450 ms.

Participants performed a same-different task on two peripheral stimuli. They were asked to press button “m” for answering ‘different’ and button “n” for answering ‘same’ as fast as they could. Target objects were abstract 3D shapes of the spiky category used by Fan et al.^1^ which have been provided to us courtesy of the authors of the original study (Fig. 1). The average size of the stimuli was 3 $\times$ 1.5$^\circ$ and their eccentricity was 7$^\circ$. The three-dimensional shapes differ from each other in four main respects: (1) the length of upper spikes, (2) the length of lower spikes, (3) the orientation of upper spikes, (4) the orientation of lower spikes (Fig. 1).

In each trial, two objects were randomly selected from the set of 1296 so that they could either be different or the same. As in the original study, for different pairs, dissimilarity was given by one or more of the 4 manipulated features. In addition, for each feature the variation between peaks occurred along multiple levels. The fixation cross was presented in the center for the duration of the experiment. At the beginning of the trial, the two targets appeared simultaneously for 100 ms on the black screen. The targets were presented in diametrically symmetric positions in opposing quadrants of the screen, alternating pseudo randomly between quadrants 1 and 3 and quadrants 2 and 4. This was made so that subjects did not have any expectations of where the targets would appear. A 7 $\times 7^\circ$ colored dynamic noise patch appeared at fixation for 83 ms at 5 stimulus-onset asynchronies (SOAs) (50, 150, 250, 350, and 450 ms). A baseline condition with no-noise was also present.

The response could be given only after the stimulus disappeared. The test was self-paced, and the next trial began 500 ms after the response button was pressed. During the practice block, visual feedback informed the participant about the accuracy of the given response. Although we did not use eye-tracking, participants were instructed to maintain central fixation. In addition, the presence of the targets on the screen was very short and would have made eye movements counterproductive^10^.

### Data analysis

Starting from the accuracy data in the peripheral same/different task, applying signal detection theory, we calculated the sensitivity index (d’) and the criterion (C). To do this we used the function dprime() from the “psycho” package^35^ which calculates d’ as the distance between the signal and signal+noise distributions with the equation d’ = z(success rate) − z(false alarms rate). The function implements the Hautus correction for extreme values, which consists of calculating the success rate as (hits + 0.5)/(hits + misses + 1), and the false alarm rate as (false alarms + 0.5)/(false alarms + correct rejections + 1)^36^. A value of 0 indicates an inability to distinguish signals from noise, whereas larger values indicate a correspondingly greater ability. The criterion is calculated as the number of standard deviations from the midpoint between the signal and signal + noise distributions, with the equation C = − (Z(hits) + Z(false alarms))/2. Negative values of C signify a bias toward responding “same’ (liberal), whereas positive values signify a bias toward responding “different” (conservative).

To test for significant differences between the SOAs, we fitted the data with a linear mixed model in which the sensitivity index (d’) was included as a dependent variable and the SOA as an ordered factor with five levels. To control for the within-subjects correlation typical of repeated measures, we also included an individual random intercept in the model. Mixed models were estimated with a Restricted Maximum Likelihood procedure (REML) with the function lmer() from the lme4 package^37^ in R. Next, we tested the fixed effects using a type III F-test with the Satterthwaite approximation method for degrees of freedom.

To assess the location of the dip, we compared each level of SOA with the baseline no-noise condition by means of five Paired samples two-tailed t-tests with False Discovery Rate (FDR) correction for multiple comparisons. The same analysis pipeline was then applied to criterion (C).

### Results

**Figure 2:**
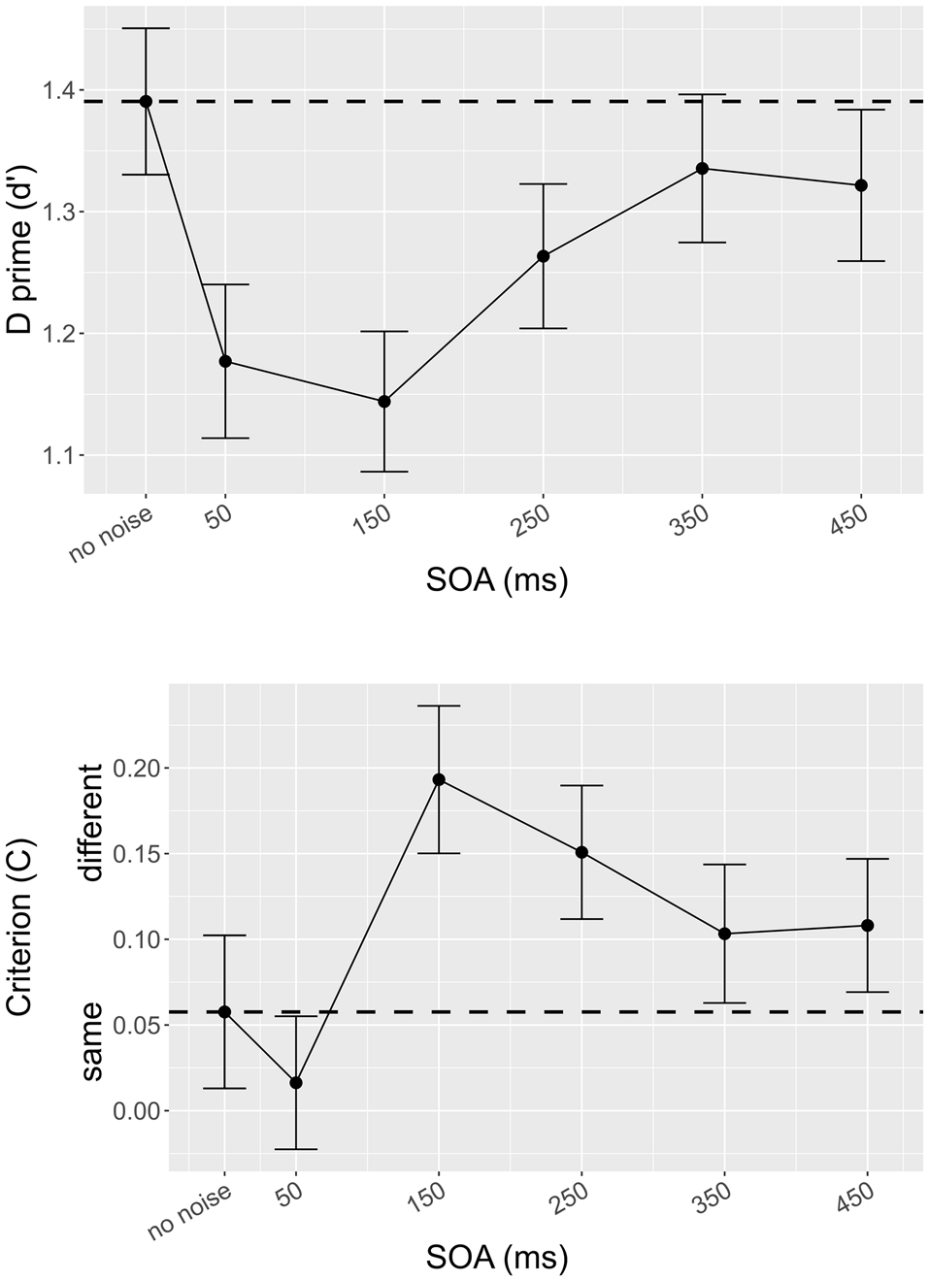
The upper panel shows d’ as a function of SOA (no-noise, 50, 150, 250, 350, 450 ms). The lower panel shows the response criterion as a function of SOA (no-noise, 50, 150, 250, 350, 450 ms). Black dots represent mean values with standard errors. The black dashed line represents the baseline no-noise condition.

The overall average d’ in this experiment was 1.248 C.I. = [1.195 1.302]. Type III F-test with the Satterthwaite approximation, with the SOA levels as the only within-subjects variable, revealed a significant main effect (F (4, 220) = 4.402; *p* = .002). Pairwise comparisons indicated that the three levels differed significantly from the no-noise condition, 50 ms (estimate = 0.214, df = 55, t = 3.98, *p* = 0.001), 150 ms (estimate = 0.247, df = 55, t = 5.28, *p* < 0.001), and 250 ms (estimate = 0.127, df = 55, t = 2.3, *p* = 0.042). On the contrary, 350 ms (estimate = 0.055, df = 55, t = 0.949, *p* = 0.347) and 450 ms (estimate= 0.069, df = 55, t = 1.218, *p* = 0.286) were not different from the baseline. This indicates a large drop in performance for SOAs lasting between 50 and 250 ms with the low point at 150 ms. d’ data are shown in Fig. 2 (upper panel).

For the criterion, mixed model analysis of variance also revealed a significant main effect for SOA (F (4, 220) = 8.154; *p* < .001). Pairwise comparisons indicated that the noise at 50 ms was not significantly different from the no-noise condition (estimate = 0.041, df = 55, t = 1.154, *p* = 0.254). Instead, 150 ms (estimate = -0.135, df = 55, t = − 4.038, *p* = 0.001) and 250 ms (estimate = − 0.093, df = 55, t = − 3.24, p = 0.005) were significantly higher than the no-noise condition. Lastly, 350 ms (estimate = − 0.045, df = 55, t = − 1.532, *p* = 0.164) and 450 ms (estimate= − 0.05, df= 55, t= − 1.732, *p*= 0.148) were not different from the baseline. This indicates a large peak in the criterion for SOAs lasting between 150 and 250 ms with the high point at 150 ms. C data are shown in Fig. 2 (lower panel).

### Discussion

The results of Experiment 1 show a decrease in sensitivity related to the presence of a foveal mask at 50, 150, and 250 ms with a global minimum at 150 ms with respect to the no-noise baseline condition. This pattern agrees with our expectations and confirms that the foveal mask presented in a time interval between 50 and 250 ms post-stimulus onset worsens performance in a peripheral visual task more than the same mask presented outside of this time window. We remotely tested a large sample of naïf participants over the internet. The number of trials per subject was smaller than in previous studies, but the total number of trials was comparable. This shows that the effect of the foveal mask is robust and it can be tested outside the laboratory. Our results are consistent with what Fan et al.^1^ found in their main experiment (see their Fig. 2). However, while our data show a single global minimum in d’ at 150 ms after stimulus onset, they found the lowest d’ at 50 ms and a second dip at 250 ms.

On the one hand, this unexpected difference could be due to some modification of the original paradigm that we were forced to make in the online adaptation. On the other hand, estimating the minimum of a function based on just 5 data points (SOA level) may be sub-optimal. With such a sparse SOA sampling, the actual minimum may fall between two SOA levels. In addition, undersampling indirectly produces a “smoothing” effect that conceals part of the variability, as well as any cyclic effects (behavioral oscillations) linked to the effect of the mask.

Regarding the criterion, we found that the foveal mask at 150 ms produces a positive shift with the participants becoming more conservative (more willing to answer “different”). A possible explanation for this result is that the foveal mask prevents a faithful reconstruction of the peripheral stimulus on the visual sketchpad.

The specific time window in which the dynamic noise mask acts on the criterion suggests that it was the mask that caused the participant to consider the two stimuli more often as different. The simultaneous presentation of the two stimuli in diametrically opposite positions excludes the many well-known stimulus-response compatibility effects^38^. However, in experiment 2, to include an additional control for unexpected effects of response mapping, we reversed the response buttons for half of the participants.

## Experiment 2

In this second experiment, we moved to the laboratory with the aim of obtaining a much better characterization of the dipper function but also verifying the presence of perceptual oscillations in performance that would predict other dips.

In the previous experiment, we found a reduction in sensitivity that ranged from 50 to 250 ms, but the SOAs were too rarefied to accurately establish the timing of the global minimum and to exclude the presence of secondary dips. In two different experiments, Fan et al.^1^ found the disruptive effect of dynamic mask at both 50 and 250 ms (see their Figs. 2 and 5). The dip at 50 ms has so far been interpreted as an attentional distraction caused by the noise onset when the peripheral stimuli are still on the screen. This explanation predicts that the two minima are caused by mechanisms that differ not only in timing, but also in nature.

However, could be that the two dips belong to one or more cyclic phenomena whose most evident expression is the minimum found at around 250 ms. If this were true, by means of a “temporally dense sampling”, we could observe the presence of these perceptual oscillations^19,27–33^.

As a first step to test this hypothesis, we analyzed the presence of one or more dips by comparing the informativeness of the linear model against that of polynomial functions of increasing degree. As a second step, based on recent studies using the same method^19,27–33^, we carefully characterize perceptual oscillations in outcomes of interest (i.e. sensitivity and criterion).

Specifically, we probed perceptual judgments of our visual stimuli at many different equally spaced SOAs, and afterward we tested the presence of significant oscillatory variations by estimating the Fast Fourier Transform (FFT) spectrum. If any peak in the FFT spectrum was statistically significant, we further evaluated the goodness-of-fit of sinusoidal fitting at the emerging frequency. The key idea of the dense sampling method is that it allows to track fluctuations in detection performance over time, allowing to evaluate the presence of perceptual oscillations defined as rhythmic variations in behavioral responses that reflect an underlying cyclic (oscillatory) neural mechanism^29,32^.

### Participants

A total of 16 participants took part in this experiment (age range: 21–36, M: 25.31, SD=3.933; 12 female). No participant fell below the removal criterion for performance, so all were included in subsequent analyses. All volunteers were students at the University of Padua and had normal or corrected-to-normal vision. They provided written informed consent before the experiments and they were compensated for their participation. The procedures were performed in accordance with the guidelines of the Ethics Committee for Psychological Research of the University of Padua, from which they were approved (prot. 3745), and in accordance with the Declaration of Helsinki.

### Apparatus

The experiment was generated using PsychoPy3^34^. Stimuli were displayed on an LCD monitor with a resolution of 1920 $\times$ 1080 pixels, at 120 Hz, and a size of 52 $\times$ 42 cm. Each participant sat in a quiet dim room, at approximately 57 cm from the screen, using a chin rest. An eye tracker (Gazepoint GP3) was used to monitor the fixation of the participants. The stimulus was not presented if participants did not look within a 2$^\circ$ radius from the fixation point. Each trial was self-initiated by button press, and after the presentation of the stimulus, participants responded by pressing the “l” or “a” as accurately as possible, with no emphasis on speed. Half of the participants pressed “l” as the “same button”, and the other half pressed “a”.

### Experimental design

The experimental design was similar to the previous one, except for the number of SOAs which were increased from 5 to 61 ranging from 0 to 500 ms, one every 8.33 ms. To allow for a robust estimate of d’, each subject performed 48 repetitions for each SOA level, while the baseline no-noise condition counted 96 trials. As the total number of trials per participant was higher than in experiment 1, we were able to reduce the sample numerosity to 16 while maintaining a comparable statistical power. The experiment started with a stimuli familiarization phase, followed by a training phase of 252 trials with feedback. The test phase consisted of three sessions of 1008 trials each, subdivided into two blocks. The participant was allowed to take a short break between blocks and a longer break between sessions. The total duration of the experiment, including breaks, was approximately three hours.

### Stimuli and procedure

The target objects were the same abstract 3D shapes as in experiment 1. The average size of the stimuli was 3 $\times 1.5^\circ$ and their eccentricity was 7$^\circ$. Eye-tracking calibration was performed before each block of trials and participants were instructed to maintain central fixation. They were informed that the next trial would not start if they moved their eyes away from the center of the screen. To prevent any bias towards one of the buttons from affecting the criterion calculation, half of the participants were asked to press the button “a” for answering "different" and the button “l” for answering "same", whereas for the other half of the participants the buttons were reversed. A 7 $\times 7^\circ$colored dynamic noise mask patch appeared in fovea for 83 ms at 61 stimulus onset asynchronies (SOAs) one each 8.33 ms ranging from 0 to 500 ms included. In the baseline condition, no-noise appeared.

### Data analysis

The sensitivity index (d’) and the criterion (C) were calculated with the same procedure as in the previous experiment. To assess the location of the dip, False Discovery Rate corrected post hoc were performed. A linear trend would indicate the absence of a dip; on the contrary, the quadratic or cubic trend would indicate its presence. To study the effect of noise as the SOA changes, we compared five different mixed models estimated with a maximum likelihood procedure (ML) with the lmer() function of the lme4 package^37^ in R. In each model, the sensitivity index (d’) appeared as a dependent variable along with a continuous variable related to the SOA. The random effect consisted of the individual intercept for the participant. In the first model, the SOA was modeled with linear regression, while in subsequent models it was modeled with polynomials of increasing order up to the fifth degree. This was done by calculating orthogonal polynomials for the SOA variable by means of the poly() function in R. The selection of the best model was carried out by considering the Akaike Information Criterion corrected for small sample sizes (AICc) which scores the models based on their log-likelihood and complexity.

Next, we performed an omnibus test (ANOVA) with Satterthwaite’s method for degrees of freedom on the selected model refitted with a REML estimation to assess the significance of the main effect of SOA. Finally, to detect the presence of the main or secondary dips, we extracted all the minima from the model fit and, for each, we extracted timing and location. We also tested whether the no-noise and the 0 ms SOA were different on average by means of a paired t-test. A similar analysis pipeline was then applied to the criterion.

Thanks to the dense sampling achieved in this experiment, we also tested the presence of behavioral oscillation in our data with the analysis used by Ronconi and Melcher in 2017^30^. First, we conducted a spectral analysis over the individual 500 ms epochs. Data were zeros-padded and Fast Fourier transformed (FFT) with MATLAB prior to statistical testing. The significance of peaks in the spectra amplitude was assessed with non parametric permutation tests. Specifically, we generated a null distribution by randomizing the SOA labels for each individual dataset, averaging the permuted datasets across subjects afterwards. We repeated this procedure 3000 times. Then the significance threshold was calculated as the 95$^\circ$ percentile of the observed amplitude values in the full set of amplitude values of the permutation distribution. Finally, as a confirmatory analysis, we performed a sinusoidal fit for each of the significant frequencies that emerged in the FFT spectrum and calculated the corresponding (observed) Adj-R^2^. For the best-fitting procedure, we applied to both the observed and permuted data a smoothing with a moving average (moving factor = 2 data points) over the zero-padded data, and the best-fit was searched in a 0.5 Hz range around the starting frequency. The procedure was significant if the observed Adj-R^2^ fell above the 95$^\circ$ percentile of the null distribution of the surrogate Adj-R^2^ derived from the permutation tests.

### Results

The overall average d’ in this experiment was 1.418 C.I.= [1.387 1.448], slightly higher than in the previous experiment. In addition to the better testing conditions, each subject performed a larger number of trials and therefore had a greater opportunity to learn. The model selection showed that the best model was the quartic model (4th-degree polynomial) which had a total predictive power of 66% compared to the full set of models as shown in Table 1.

**Table 1:** Model selection table for d’.

Model selection based on AICc				
Modnames	K	AICc	Delta AICc	AICc weight
quartic	7	985.07	0.00	0.66
quintic	8	987.10	2.03	0.24
cubic	6	988.94	3.87	0.10
linear	4	999.61	14.55	0.00
quadratic	5	1000.68	15.62	0.00

The best-fit model is listed first. K, the number of parameters in the mixed model including fixed and random effects. AICc, Akaike Information Criterion corrected for small sample sizes, the smaller the AIC value, the better the model fit. Delta AICc, the difference in AIC score between the best model and the model being compared. AICc weight, which is the proportion of the total amount of predictive power provided by the full set of models contained in the model being assessed.

**Figure 3:**
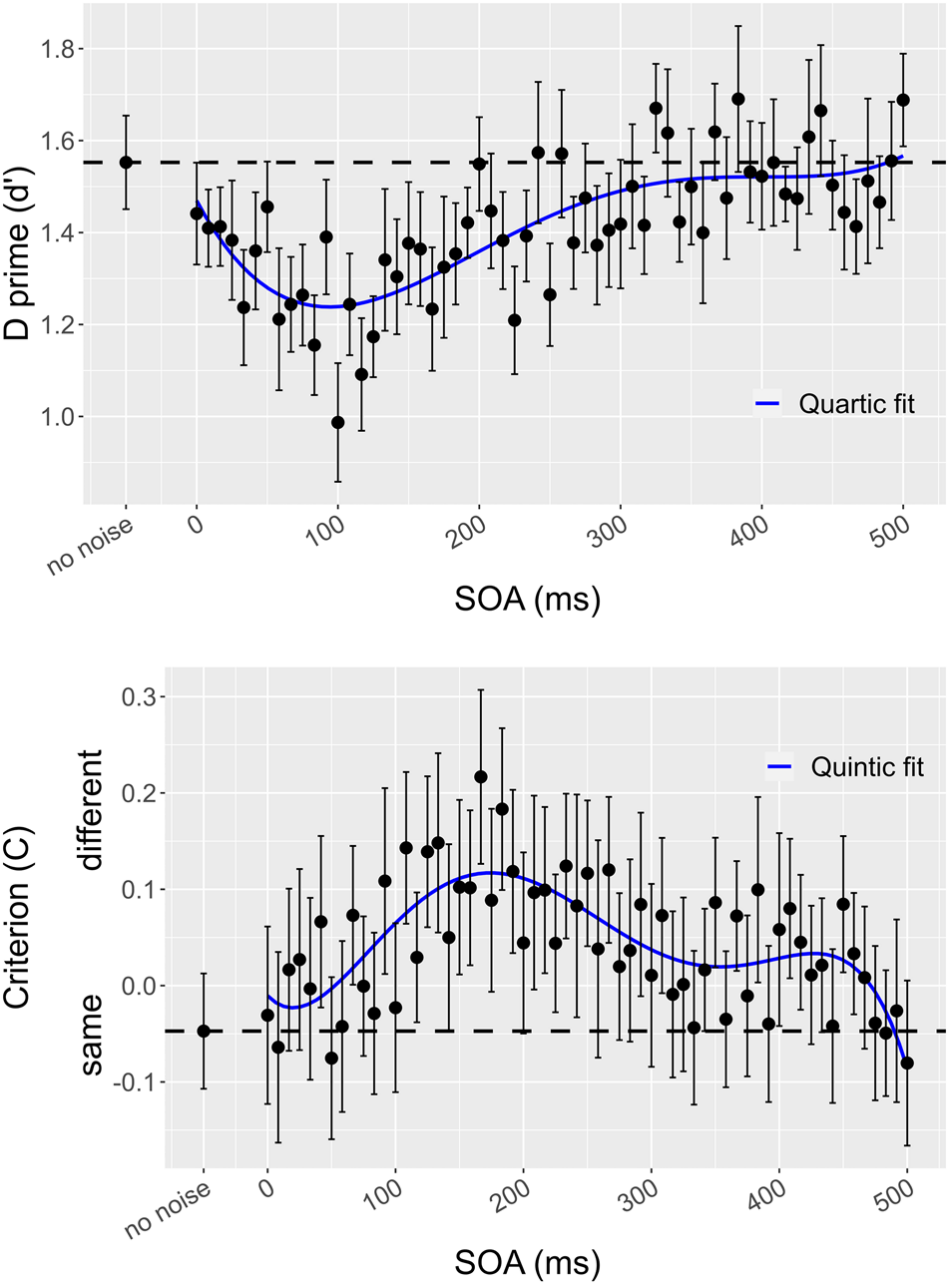
The upper panel shows d’ as a function of SOA. The lower panel shows the response criterion as a function of SOA. Black dots represent mean values with standard errors. The black dashed line represents the baseline no-noise condition. The solid blue line represents the polynomial fit.

The type III F-test with the Satterthwaite approximation revealed a significant main effect of the 4th-degree polynomial predictor SOA (F (4, 956) = 19. 445; *p* < .001). The quartic model predicts a global minimum that was located at a d’ of 1.24 and 94 ms after stimulus onset. Furthermore, there was no significant difference between the condition without noise and the condition with concomitant noise (estimate = 0.111, df = 15, t = 1.259, *p* value = 0.228) or the condition with noise onset at 500 ms after the target onset (estimate= − 0.136, df = 15, t = − 1.594, *p* value = 0.228). d’ data are shown in Fig. 3 (upper panel).

For the criterion, the model selection showed that the best model was the quintic (5th-degree polynomial) which had a total amount of predictive power of 96% compared to the full set of models as shown in Table 2.

**Table 2:** Model selection table for the criterion. The best-fit model is listed first. K, the number of parameters in the mixed model including fixed and random effects. AICc, Akaike Information Criterion corrected for small sample sizes, the smaller the AIC value, the better the model fit. Delta AICc, the difference in AIC score between the best model and the model being compared. AICc weight, which is the proportion of the total amount of predictive power provided by the full set of models contained in the model being assessed.

Model selection based on AICc				
Modnames	K	AICc	Delta AICc	AICc weight
quintic	8	− 250.90	0.00	0.96
cubic	6	− 243.66	7.25	0.03
quartic	7	− 242.27	8.64	0.01
quadratic	5	− 239.96	10.95	0.00
linear	4	− 212.02	38.89	0.00

Mixed model analysis of variance revealed a significant main effect of the 5th-degree polynomial predictor SOA (F (5, 955) = 10.231; *p* < .001). The quintic model predicts a global maximum that was located at a C value of 0.117 and 174 ms after stimulus onset, Furthermore, there was no significant difference between the condition without noise and the condition with concomitant noise (estimate = − 0.0165, df = 15, t = − 0.285, *p* value = 0.78) or the condition with 500 ms noise (estimate = 0.033, df = 15, t = 0.524, *p* value = 0.78). C data are shown in Fig. 3 (lower panel).

#### Spectrum analysis and best fit

**Figure 4:**
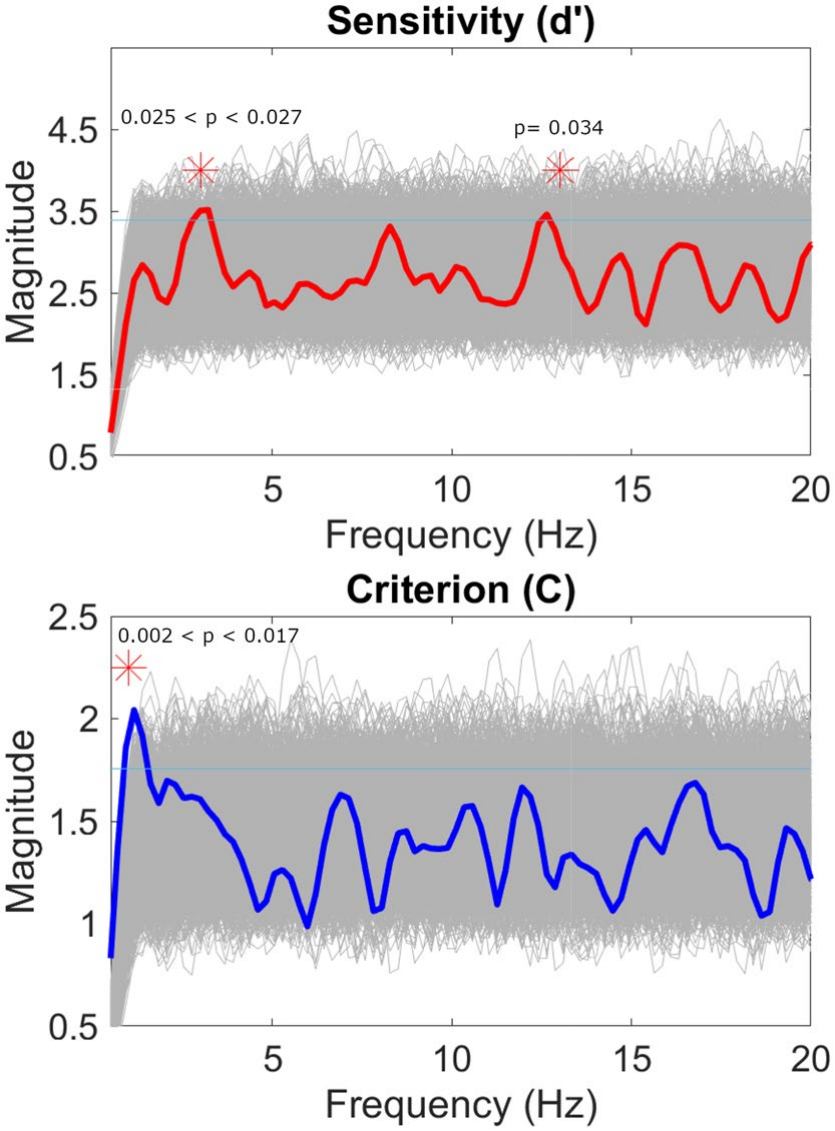
Power spectrum analysis for d’ (upper panel) and criterion (lower panel). The horizontal line shows the significance threshold, frequencies that cross the line are statistically different from chance.

The power spectrum analysis for d’ showed a peak at 3 Hz, with observed values significantly higher than the permutation spectrum in the 2.989–3.218 Hz frequency range (0.025 < p < 0.027). A second significant peak was found at 12.644 Hz (*p* = 0.034). For the C, we observed a single peak at 1 Hz, with observed values significantly higher than the permutation spectrum in the 0.92–1.379 Hz frequency range (0.002 < p < 0.017). Results from the power spectrum analysis are shown in Fig. 4.

**Figure 5:**
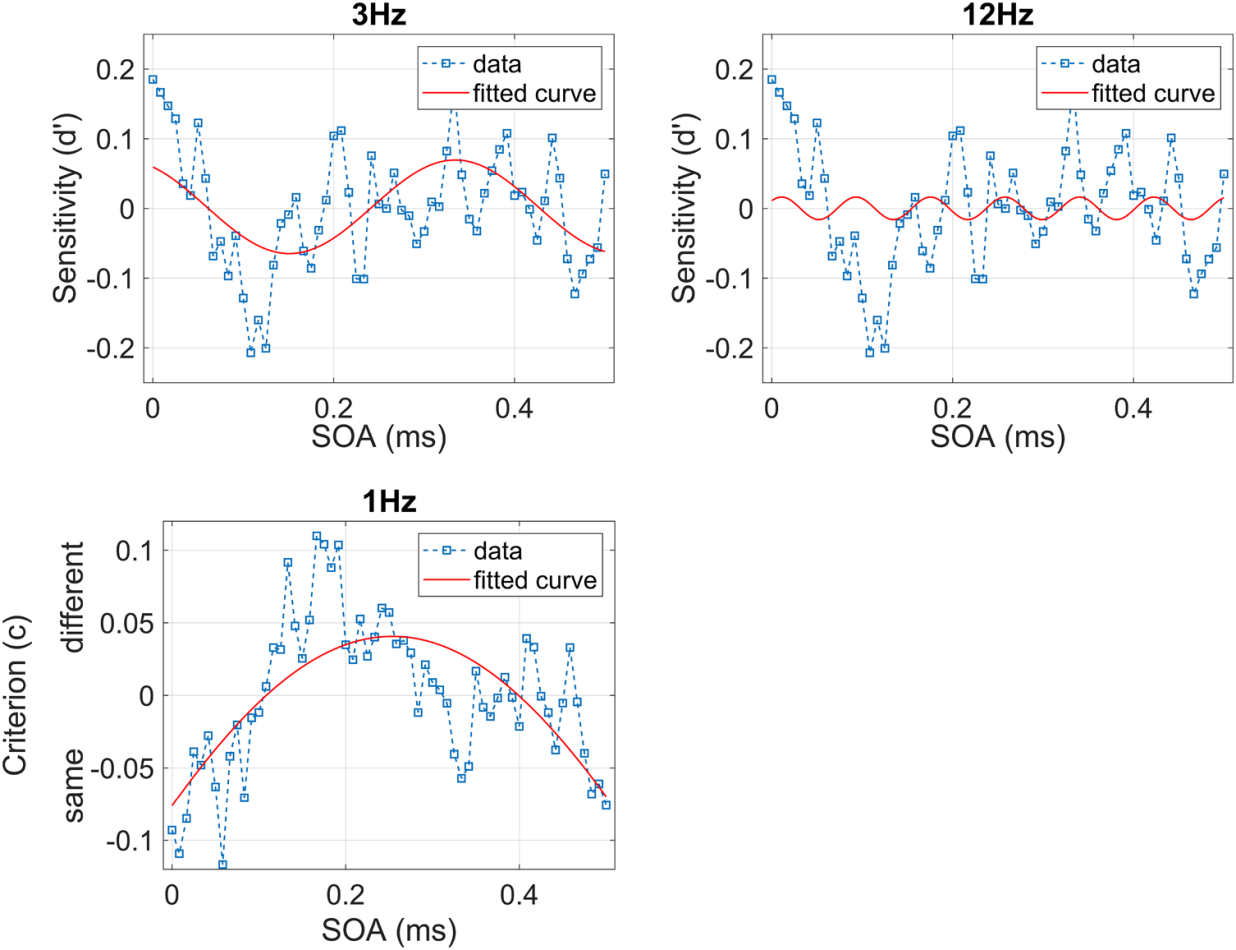
The upper panel shows the 3 Hz (upper left panel) and 12 Hz (upper right panel) sinusoidal fit for d’. The lower panel shows the 1 Hz sinusoidal fit for criterion.

The best-fitting procedure for the d’ revealed that, for 3 Hz frequency, the observed Adj-R2 was significant (Adj-R^2^ = 0.24, *p* = 0.019, best-fitting frequency = 2.727 Hz, C.I. = [2.306, 3.147]). However, it was not significant for 12 Hz (Adj-R^2^ = − 0.016, *p* = 0.866, best-fitting frequency = 12.14 Hz, C.I. = [fixed at bound]). For the C, the best-fitting procedure revealed that the observed Adj-R2 for the 1 Hz was significant (Adj-R^2^= 0.454, *p* < 0.001, best-fitting frequency = 0.65 Hz, C.I. = [− 3.071, 4.371]). Results from the best-fit analysis are shown in Fig. 5.

### Discussion

In this experiment, dense sampling of the SOA allowed us to accurately estimate the temporal characteristics that best describe the effect of noise with respect to sensitivity and criterion. Regarding sensitivity, we were able to confirm the presence of a global dip at about 100 ms after stimulus onset. This timing was consistent with that found in Experiment 1 (150 ms) and earlier than that found in the original study (250 ms)^1^.

Judging from our results, in the previous literature, the chosen intervals were not ideal for precisely estimating the global minimum in sensitivity. By using sparse sampling (generally 5 levels of SOA^1^), you may measure a biased timing of the dip which ends up falling into the nearest SOA level. Here, we have provided a detailed characterization of the time course, which may help to estimate suitable levels for future studies. We also note that in experiment 2, simultaneous or highly delayed noise (>450 ms) has no effect on sensitivity. Therefore, we found no evidence for the presence of two dips within the first 200 ms contrary to what was reported by Fan et al.^1^.

Regarding the criterion, we confirmed the presence of a maximum at about 174 ms, indicating more conservative responses. It is also important to note that there was a delay of about 80 ms between the minimum in d’ and the maximum in C. This suggests that the foveal mask acts on at least two separate mechanisms that influence sensitivity and criterion at different times. This result is further supported by frequency spectrum analysis. Although sensitivity is affected by noise cyclically at 3 and 12 Hz, for the criterion the emerging frequency was 1 Hz. Further confirmation of this comes from the best-fitting analysis. The sinusoidal fit was significantly different from chance at 3 Hz for sensitivity and at 1 Hz for criterion. The fact that the sinusoidal fit at 12 Hz was not significant does not invalidate the result of the FFT analysis. Indeed, this latter analysis is not only sufficiently conservative but also more sensitive to the coexistence of multiple frequencies in the data; with the sinusoidal fitting, on the contrary, higher frequencies might be more penalized because they are more affected by noise in the data.

Overall, our data strongly suggest the existence of two distinct mechanisms influencing sensitivity and criterion response. Thus, we can infer that foveal feedback interferes with incoming feedback to early visual areas from diverse sources (and thus with different timing). We can imagine the existence of at least two different feedback sources, one perceptual in origin and the latter more cognitive.

## General discussion

Recent studies have pointed to the possible role of the foveal retinotopic cortex in the elaboration of peripheral information, presumably via a temporally flexible feedback signal from the higher-level cortex to the lower-level foveal cortex^1,6,10–13^. These studies show that processing foveal visual stimuli in a narrow time window shortly after peripheral stimulus presentation interferes with peripheral object discrimination. Consistent with this finding, Fan et al.^1^ found that presenting a foveal mask 250 ms after the stimulus onset disrupts discrimination performance in the periphery. This effect primarily occurs for tasks that involve spatial details, providing “psychophysical evidence that the high-resolution specialization of the foveal cortex can be used for discriminating fine spatial details of peripheral objects” (p. 11631)^1^.

Experiment 1 was able to reproduce the non monotonic SOA function observed in the original experiment by Fan et al.^1^. In our data, we could identify a clear drop in sensitivity when the noise was presented around 150 ms after stimulus onset. We have also shown that foveal mask interferes with the decision-making process, altering the response criterion in a more conservative direction. These effects were sufficiently robust to be tested online in non expert participants but the timing of the dip in the d’ differed by about 100 ms from what Fan found in 2016^1^. It is true that Fan et al.^1^ found that the timing for noise interference can vary depending on the amount of mental manipulation required on peripheral objects to solve the task. However, since we used the same task and stimuli as in the main experiment of the original study, this explanation does not apply to our case. This discrepancy in timing led us to perform a second experiment to measure more accurately the sensitivity (d’) as a function of noise. To do this, we increased the levels of SOAs to better sample the time course of the effect.

Experiment 2 again supports the hypothesis that foveal noise interferes with a peripheral task when the noise is presented in a narrow time window. However, as in experiment 1, we found that the strongest masking was earlier than 250 ms, specifically at 150 ms in experiment 1 and 94 ms in experiment 2. Although it was still consistent with the foveal feedback hypothesis, the timing found in our second experiment precedes that of other studies conducted with similar paradigms. In two different studies, Weldon et al. found a dip in performance at 117 ms^10,11^. Yu et al. found the dip at 150 ms^12^, while Ramezani et al. found the dip at 300 ms^13^. There are multiple causes for this variability across studies. In part this was due to diversity in the stimuli and tasks, but mostly this variability could be attributed to the use of a limited number of SOAs. Only a few SOA levels (3 to 5) were used in previous studies, and the dip was localized through pairwise comparisons between different SOA levels, without any interpolation. Instead, through dense sampling, we were able to estimate the function that best describes the effect of the mask and then use this function to find a minimum.

Given the high individual variability in the observed phenomenon, the low sample size of previous studies may also have contributed to the differences in the findings. In our study, we mitigated this problem by using a large sample size (experiment 1) and a large number of trials (experiment 2). TMS-based studies have also suffered from these limitations^7,9^. In addition, it is not possible to make a direct comparison between TMS and masking techniques, as the difference between the two procedures can lead to differences in timing. While the TMS stimulus directly activates the cortex, the noise mask activates the entire visual pathway starting from the retina before interfering with the cortical process. Moreover, unlike a mask, the TMS stimulus interferes instantaneously with neural activity. Our results, therefore, do not necessarily contradict what has been reported in previous studies, but rather they estimate more accurately the temporal dynamics of the disruptive effect of the mask on the d’.

We also confirmed an effect of noise on the criterion, which, as in the first experiment, becomes more conservative, but with a different timing with respect to the sensitivity. This suggests that there are two distinct processes, one of a more perceptual nature and one more cognitive. We have also verified the co presence of cyclical phenomena involving the sensitivity, one at a frequency close to 3 Hz and the other at a higher frequency, close to 12 Hz. These additional data suggest that the dip in sensitivity produced by the foveal mask could arise as a combination of rhythmic variations at slower and faster frequencies, with faster perceptual oscillations reflecting the basic sampling frequency of the visual system within the alpha band and the slower rhythm possibly resulting from the extended network encompassing associative and control areas outside the visual system^23,25,39^. The criterion (C) seemed to show an oscillatory pattern at 1 Hz, although the sampling interval in our experimental design allowed us to measure only about half of a cycle at this frequency, so this result should be taken with caution. It is possible that the foveal mask interferes with task execution at distinct stages; the first, earlier, related to the perceptual representation of the stimulus, and the second, slower, linked to the decision process. Imaging studies showed that perception and imagery rely on similar neural representations throughout the ventral visual stream and that in retinotopic visual areas representational similarity is greater when the task requires processing of visual details^40^. In re-imagining the stimulus to solve a discrimination task, it might be convenient to discard positional information. This could lead to the post stimulus foveal activation found by Williams and colleagues in their seminal study^6^.

The relationship between foveal feedback and mental imagery is also supported by studies showing the involvement of the foveal cortex in processing tactile stimuli^41^. Evidence that a TMS pulse in V1 in the range of 120 to 220 ms reduces performance in a tactile Braille letter recognition task^42^ suggests that foveal feedback is not specific to the visual modality, but instead is part of a general mechanism activated whenever high-resolution buffers are needed for geometric calculations and object processing^43^. We therefore observe a strong similarity between the foveal feedback and the visuospatial sketchpad theorized in Baddeley and Hitch’s tripartite model of working memory^44^. In their model, the sketchpad, similar to an inner eye, allows individuals to revisit mental images pertinent to the task at hand. Baddeley ^17^ theorized that the sketchpad is the storage place in working memory that contains the necessary object information to set up and manipulate mental visual images. Although with different connotations, such a construct is also present in Kosslyn’s definition of surface and deep representation^18^.

As is known, perception and imagination share neural representations in the alpha frequency band and the contents of these shared representations are likely to be complex visual features^45^. The fact that noise impacts sensitivity with a cyclic rate might be an indication that it interferes with the cyclic endogenous feedback system in general, but the global minimum at about 100 ms shows that this interference is greater when the feedback carries image information critical to task execution^26^. Is there a more parsimonious explanation for this phenomenon? In the reverse hierarchy theory, shape discrimination involves an initial feedforward sweep followed by feedback for finer shape processing^46^. Moreover, behavioral data from perceptual learning studies show that information from low-level retinotopic and high-level non retinotopic areas may be combined to support object recognition^47^. However, there are three features that make foveal feedback a special case. First, this feedback has an impact during the current trial response and thus it is not related to a long-term perceptual fine-tuning or learning. Second, the feedback is not directed to the retinal areas where the stimuli are presented, but instead is position-invariant. Third, despite the fact that a reverse hierarchy model may imply a time course^46^, we would expect a detrimental effect of noise at all SOAs. Instead, the maximum effect of noise is found when it appears after the offset of the targets. Therefore, it is more plausible that this feedback is part of a system of mental image formation.

## Conclusion

In this study, we extended previous findings about the temporal dynamics of foveal feedback. We showed that the dip in sensitivity is earlier than previously found and with no obvious secondary peaks. Moreover, for the first time, we show the presence of a change for the decision criterion (response bias) that is temporally distinct from that of sensitivity. The fact that the effect of noise on the criterion is shifted in time with respect to sensitivity suggests that noise interferes with feedback from higher areas at several levels and with different timing. Finally, we found evidence for perceptual oscillations in the disruptive effect of the mask effect on sensitivity at both 3 and 12 Hz. These results suggest that the foveal mask affects two mechanisms, one more perceptual and faster and one more cognitive and slower.

## Data Availability

The experimental design and analyses of the first experiment were pre-registered on Open Science Framework (OSF) before data collection. The datasets analyzed during the current study are also available in the OSF repository.
